# Both seed germination and seedling mortality increase with experimental warming and fertilization in a subarctic tundra

**DOI:** 10.1093/aobpla/plx040

**Published:** 2017-09-01

**Authors:** Ann Milbau, Nicolas Vandeplas, Fred Kockelbergh, Ivan Nijs

**Affiliations:** 1 Research Institute for Nature and Forest (INBO), Kliniekstraat 25, 1070 Brussels, Belgium; 2 Research Group of Plant and Vegetation Ecology (PLECO), Department of Biology, University of Antwerp, Universiteitsplein 1, 2610 Wilrijk, Belgium

**Keywords:** Abisko, arctic, *Betula nana*, climate change, fertilization, germination, range expansion, seedling establishment, tundra, *Vaccinium myrtillus*

## Abstract

Climate change is expected to force many species in arctic regions to migrate and track their climatic niche. This requires recruitment from seed, which currently shows very low rates in arctic regions, where long-lived and vegetatively reproducing plants dominate. Therefore, we pose the question whether recruitment (germination and seedling establishment) in arctic regions will significantly improve in a warmer world, and thus allow species to follow their climatic niche. We used a full factorial experiment to examine if realistic warmer temperatures (+3 °C; infrared radiation) and increased nitrogen availability (+1.4 g N m^−2^ year^−1^) affected germination, seedling survival and above- and below-ground seedling biomass in five species common in subarctic regions (*Anthoxanthum odoratum*, *Betula nana*, *Pinus sylvestris*, *Solidago virgaurea*, *Vaccinium myrtillus*). We found that warming increased seedling emergence in all species, but that subsequent mortality also increased, resulting in no net warming effect on seedling establishment. Warming slightly increased above-ground seedling biomass. Fertilization, on the other hand, did not influence seedling biomass, but it increased seedling establishment in *B. nana* while it reduced establishment in *V. myrtillus*. This may help *B. nana* dominate over *V. myrtillus* in warmer tundra. Surprisingly, no interactive effects between warming and fertilization were found. The lack of a general positive response of seedling establishment to warmer and more nutrient-rich conditions suggests that (sub)arctic species may experience difficulties in tracking their climatic niche. Predictions of future species distributions in arctic regions solely based on abiotic factors may therefore overestimate species’ ranges due to their poor establishment. Also, the opposite response to fertilization of two key (sub)arctic dwarf shrubs, i.e. *B. nana* and *V. myrtillus*, could have important implications for the future development of arctic plant communities and argues for more research into the role of fertilization for plant establishment.

## Teaser

Climate change is expected to cause (sub)arctic plant species to move polewards to track their climatic niche. However, rapid migration requires recruitment from seed, which is rare in arctic regions where most plants reproduce vegetatively. Here, we examined whether recruitment from seed would improve under warmer and more fertile future conditions. We found that seedling establishment was little affected by warming and fertilization, suggesting that (sub)arctic species may experience difficulties in tracking their climatic niche. Predictions of future species distributions in arctic regions solely based on abiotic factors may therefore overestimate species’ ranges.

## Introduction

Arctic ecosystems have warmed by between 1 and 4 °C since 1960, at a substantially higher rate than other biomes (Hansen *et al.* 2010; Serreze and Barry 2011), and more rapid warming than the global mean is predicted to continue in the Arctic during this century (IPCC 2013). This forces many plant species to adapt to their changing environment, or to migrate and track their climatic niche (Graae *et al.* 2009; Shevtsova *et al.* 2009; Müller *et al.* 2011). These processes require sexual reproduction, whereas arctic ecosystems are dominated by long-lived and vegetatively reproducing plants (Billings and Mooney 1968; Bell and Bliss 1980; Totland 1997). Therefore, a key question is whether recruitment from seed, which is essential for rapid migration, will significantly improve under future environmental conditions in cold-climate regions.

While several studies indicate that temperature increases of 1–3 °C may positively influence a variety of reproductive parameters in plants from arctic regions, such as flowering phenology, flower biomass, seed production and seed viability (Wookey *et al.* 1994, 1995; Alatalo and Totland 1997; Molau 1997; Molau and Shaver 1997; Welker *et al.* 1997; Gugerli *et al.* 2008; Gugerli and Bauert 2011; Klady *et al.* 2011), it is less clear how seed germination and seedling establishment will respond to a warmer climate. Germination and seedling establishment are considered major bottlenecks in arctic plant life history (Chambers and MacMahon 1994; Clark *et al.* 2007; Shevtsova *et al.* 2009; Graae *et al.* 2011) and show very low rates under field conditions. For instance, in Canadian high-arctic tundra first-year germination of 10 species was only 4 % on average (Bell and Bliss 1980), and in subarctic Sweden average seedling emergence of 17 species was 7.5 %, and their subsequent mortality rate 80 % (Milbau *et al.* 2013). Graae *et al.* (2011) observed germination rates of 0.9 % in undisturbed and 11 % in disturbed subarctic tundra vegetation, averaged over 10 species, and under high-arctic conditions on Svalbard germination in outdoor conditions was generally below 5 %, compared to *c.* 80 % under optimal lab conditions for the same seed source (Müller *et al.* 2011). These low success rates are often attributed to low temperatures, low nutrient availability and a short growing season (Billings and Mooney 1968; Bell and Bliss 1980; Molau and Larsson 2000), although also other abiotic (e.g. water, light, soil structure) and biotic (e.g. competitors, pathogens, predators) factors are known to influence the post-dispersal establishment of seedlings (Fenner 2000; Clark *et al.* 2007; Klanderud 2010; Eckstein *et al.* 2011; Graae *et al.* 2011; Müller *et al.* 2011; Soudzilovskaia *et al.* 2011; Milbau *et al.* 2013).

Climate warming is expected to alleviate some of the physiological constraints seeds and seedlings experience in cold environments, but knowledge about the role of climate warming for germination and seedling survival in arctic species solely derives from controlled lab studies, or outdoor studies on bare soil (but see Hobbie and Chapin 1998). Most lab studies thus far have indicated improved germination of (sub)arctic seeds under warmer conditions (Bell and Bliss 1980; Graae *et al.* 2008; Milbau *et al.* 2009; Müller *et al.* 2011), although the applied warming has often been more than what can be expected by climate change. Field studies on bare soil, on the other hand, showed a reduction in recruitment success due to warming. For instance, Graae *et al.* (2009) found reduced germination and seedling establishment in *Polygonum vivparum* and *Saxifraga cernua* when exposed to elevated temperatures (+2 to +8 °C), and also Shevtsova *et al.* (2009) observed that warming with 3 °C reduced seedling establishment in several important subarctic species. Because of the bare soil, this could have been caused by heat stress (Graae *et al.* 2009). In contrast, the only study we know of that studied germination in intact arctic plant communities showed that air warming by 1 °C doubled the amount of germination in five tree species (Hobbie and Chapin 1998). Recent seed addition studies using natural temperature and precipitation gradients in Southern Norway, in alpine systems largely comparable to our subarctic study site, indicated higher species emergence of both trees (Tingstad *et al.* 2015) and alpine species (Klanderud *et al.* 2017) in cold alpine than in warmer subalpine and boreal sites, suggesting that low temperatures were not limiting recruitment. However, it should be noted that in gradient studies like these, the effect of temperature cannot be separated from other co-varying factors.

Next to temperature, also the availability of nutrients is expected to increase in arctic regions. This is due to faster mineralization of soil organic matter in a warmer climate (Rustad *et al.* 2001; Buckeridge and Grogan 2008; Baptist *et al.* 2010) and increased atmospheric nitrogen deposition (Langner *et al.* 2005; Kühnel *et al.* 2011, 2013). Low nutrient levels have been shown to limit plant reproduction (Wookey *et al.* 1995) and the growth and survivorship of seedlings (Hobbie and Chapin 1998) in tundra sites. For instance, fertilization increased flower density, number of seeds per flower and seed weight in *Dryas octopetala* on Svalbard (Wookey *et al.* 1995), and likewise improved the growth of seedlings of *Betula glandulosa* at the forest-tundra ecotone (Paradis *et al.* 2014). In addition, nitrogen and especially nitrate are known to stimulate germination in a variety of species (Bewley and Black 1982; Hilhorst and Karssen 1992; Baskin and Baskin 1998), and some species are even absolutely dependent on the presence of nitrate to germinate (Bouwmeester *et al.* 1994). It is, however, unclear whether nitrogen also improves germination and seedling survival in plants growing in nutrient-poor arctic environments. Also, the interactive effects of nitrogen addition and warming on early plant life stages in arctic environments remain to be examined.

In the current study, we explore how two important components of global change, warmer temperatures and increased nitrogen availability, influence seedling emergence, growth and establishment in subarctic tundra in northern Sweden. Specifically, we added five species common to the subarctic, as seeds and as pre-grown seedlings, to extant tundra communities and examined the effects of warming (infrared radiation, Free Air Temperature Increase [FATI]) and fertilization and their interactions on germination (amount and timing), seedling survival and above- and below-ground seedling biomass. Because of the generally positive effects of temperature and nitrogen on germination and plant growth (Hilhorst and Karssen 1992; Parsons *et al.* 1994; Baskin and Baskin 1998; Arft *et al.* 1999; Probert 2000), we expect germination and seedling establishment to improve under a realistic scenario of warmer and more nutrient-rich conditions in subarctic tundra. We also expect fertilization to reinforce the positive effect of warming on seedling growth, because increased plant biomass in response to warming should increase plant nitrogen demand (An *et al.* 2005). By using the FATI technique, we could test our hypotheses under strictly controlled temperature regimes in identical plant communities, thereby excluding confounding factors as opposed to most other types of outdoor warming studies (i.e. open top chambers or natural gradient studies).

## Methods

### Site description

The study was performed in a typical subalpine tundra site at 418 m a.s.l. in Abisko, Swedish Lapland (68°21′N, 18°49′E). The climate in the region is subarctic montane, with a growing season length of *c.* 3 months, from mid-June to early-mid September (Molau *et al.* 2005). Climate data from the nearby Abisko Scientific Research Station (385 m a.s.l.; 1961–90) indicate a mean annual temperature of −0.8 °C and a mean July temperature of 11 °C. Average annual precipitation (1980–99) is 304 mm, of which approximately one-third falls during the summer. Common vascular plant species at the study site were *Empetrum hermaphroditum*, *Vaccinium uliginosum*, *Carex bigelowii*, *Andromeda polifolia* and *Rhododendron lapponicum*. The soil was a gelic gleysol with a well-developed, at least 10 cm deep, humus layer on a bedrock of base-rich mica schist.

### Study species and plant material

We selected five species: *Anthoxanthum odoratum* (grass), *Betula nana* (dwarf shrub), *Pinus sylvestris* (tree), *Solidago virgaurea* (forb) and *Vaccinium myrtillus* (dwarf shrub). They represent a range of functional types, are known to respond to warming (Parsons *et al.* 1994; Walker *et al.* 2006; Milbau *et al.* 2009; Shevtsova *et al.* 2009) and are important components of subarctic plant communities. The dwarf shrubs included an early (*B. nana*) and a late (*V. myrtillus*) germinating species (Milbau *et al.* 2009). All species are abundant in the study area, apart from *P. sylvestris*, which occurs sporadically, but is expected to expand its range to more northern latitudes (Matías and Jump 2014).

We used both seeds (‘seed sowing study’) and pre-grown seedlings (‘seedling planting study’) to test how different early life stages were affected by warming and nitrogen addition. Due to space restrictions related to the use of the infrared warming technique, germination could only be studied in three out of the five species. Seeds for the seed sowing study (*B. nana*, *S. virgaurea* and *V. myrtillus*) were collected between 15 August and 15 September 2008 in the Abisko area (68°21′N, 18°49′E), stored dry for *c.* 4 months and then stratified for 20 weeks on wet filter paper at 0.5 °C until sowing. For the seedling planting study, the same seed sources as for the seed sowing study were used. Additionally, seeds of *A. odoratum* were collected in the Abisko area in autumn 2008 and seeds of *P. sylvestris* were ordered from a seed company in Karesuando (68°20′N, 21°53′E), thus originating from a similar latitude. From 15 October 2008, the seeds were stratified on moist filter paper at 0.5 °C for 20 weeks. Afterwards, they were put in temperatures of 20/10 °C 12/12 h to stimulate germination and once germinated they were planted in meadow soil collected in the study area, and grown for 3 months at 18/10 °C 12/12 h. Before planting them in the outdoors experiment (7 June), the length of the three largest leaves on each individual was measured as a non-destructive measure for initial plant size.

### Experimental design

**Figure 1.**
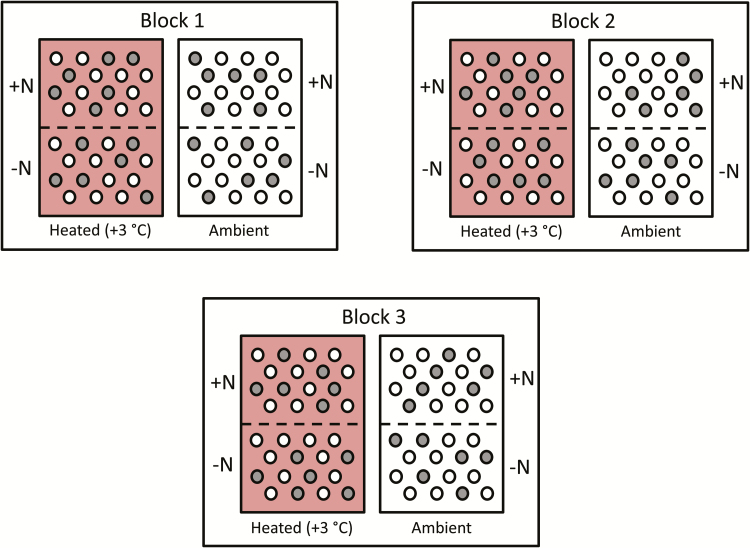
Overview of the experimental design. The experiment consisted of three pairs (blocks) of plots, of which one plot per pair was assigned to a warming treatment (+3 °C) by means of infrared irradiation. Each plot was further divided into two subplots by a Plexiglass plate (dashed line), and one subplot per plot was assigned to a nitrogen addition treatment (‘+N’: fertilized with N; ‘−N’: no fertilizer added). Each subplot contained 16 3-cm diameter gaps, evenly distributed over the area. We assigned two gaps per species (*Betula nana*, *Solidago virgaurea* and *Vaccinium myrtillus*) to the seed sowing study (‘seed sowing gaps’; grey) and two gaps per species (*Anthoxanthum odoratum*, *B. nana*, *Pinus sylvestris*, *S. virgaurea* and *V. myrtillus*) to the seedling planting study (‘seedling gaps’; white). At the start of the experiment, we added 30 seeds of the assigned species to each seed sowing gap and we planted one pre-grown seedling in each seedling gap.

We selected three pairs (blocks) of plots with similar species composition in meadow vegetation, and per pair we assigned one plot to an ambient and one to a warming (+ 3 °C) treatment (Fig. 1). Each plot was 40 × 50 cm and 2 m apart from the other plot in the same pair. The different pairs were at least 10 m apart. Each plot was further split into two subplots (each 40 × 25 cm) of which one was assigned to a control and one to a nitrogen addition treatment. The division was made by a Plexiglass plate, inserted in the soil to a depth of 15 to 20 cm to prevent the fertilizer from spreading into the adjacent subplot. The majority of roots in the study area are situated in the upper 10 cm of soil (Jackson *et al.* 1996; Iversen *et al.* 2015). Species composition and cover in each subplot were estimated on 24 July.

Within each subplot we created 16 small gaps of 3 cm diameter, evenly dispersed over the subplot surface. We randomly assigned 10 gaps to the five species used in the seedling planting study (two gaps per species; hereafter ‘seedling gaps’). The other six gaps were assigned to the three species of the seed sowing study (two gaps per species; hereafter ‘seed sowing gaps’). On 7 June, one seedling was planted per seedling gap and 30 seeds were added to each seed sowing gap.

### Heating treatment

Heating was achieved under field conditions in the absence of enclosure (e.g. open top chambers), using the FATI technique (Nijs *et al.* 1996, 2000). Each of the three heated plots was equipped with a set of irradiation sources, suspended above the plot from the north side and irradiating the plots by a computer-controlled, modulated flux density of infrared radiation (0.8–3 μm). The equipment was set to yield a continuous increase of the vegetation surface temperature of ~3 °C above ambient. Each of the three control plots served as a system control and had a ‘dummy’ FATI unit without lamps, to create similar obstruction of radiation when the sun was in the north. Surface temperature was measured in each plot with non-contact semiconductor sensors (‘infracouple’, type OS39-MVC-6; Omega Engineering, Stamford, CT, USA). Additionally, air temperature at 5 cm above the soil surface and soil temperatures at 2.5, 7.5 and 10 cm depth were measured in each plot with NTC-thermistors (EC95; Thermometrics, Edison, NJ, USA). These data were recorded every 30 min (DL2E data logger; Delta T, Cambridge, UK). Soil volumetric water content in the top 5 cm soil layer was measured every minute and mean values per hour were stored on an hourly basis (EC-5 soil moisture sensors and Em50 data loggers; Decagon Devices, Pullman, WA, USA). The heating treatment started on 8 June and ended on 21 August 2009, which represents the total duration of the experiment.

### Nitrogen addition treatment

We added fertilizer to an amount of 1.4 g N m^−2^ year^−1^. This amount was chosen to represent realistic future levels of N in subarctic tundra ecosystems as a result of increased mineralization (Zamin and Grogan 2012) and elevated atmospheric N deposition (Granath *et al.* 2009). We added fertilizer on four occasions: 8 June, 30 June, 20 July and 11 August by adding each time 0.35 g N m^−2^ as NH_4_NO_3_ in aqueous solution (2.5 L water per m^2^ of fertilized area). The same amount of water was added to the non-fertilized subplots.

On 8 June, we inserted three resin capsules (PST-1; Unibest, Bozeman, MT, USA) per subplot to test if the fertilization treatment was successful in increasing the bioavailable amounts of NO_3_^−^ and NH_4_^+^, and if heated and non-heated plots differed in nutrient availability. The capsules were retrieved at the end of the experiment, on 21 August, and were extracted by shaking them three times for 30 min with 10 mL of a 2 M KCl solution (30 mL of KCl in total). The KCl extractable concentrations of NO_3_^−^ and NH_4_^+^ were subsequently determined by means of flow injection analysis (FIAstar 5000; FOSS NIRSystems, MD, USA).

### Plant measurements

The number of emerged seedlings and their survival, in addition to the survival of the transplanted seedlings, were recorded every fifth day throughout the duration of the experiment (8 June until 21 August).

The calculated recruitment data for the seed sowing study were: total seedling emergence proportion (cumulative number of emerged seedlings/number of sown seeds; referred to as ‘seedling emergence’ hereafter), seedling mortality proportion (number of dead seedlings/number of germinated seeds; referred to as ‘seedling mortality’ hereafter) and seedling establishment proportion at the end of the experiment (number of survived seedlings/number of sown seeds; referred to as ‘seedling establishment’ hereafter). To study the effects of the treatments on speed of germination, we estimated mean germination time (MGT), which was calculated as:

$$MGT=\sum_{1}^{i}n_{i}t_{i}/N$$

with *n*_*i*_ the number of seeds that germinated within consecutive intervals of time, *t*_*i*_ the time between the beginning of the test and the end of a particular interval of measurement, and *N* the total number of seeds that germinated (Deines *et al.* 2007; Milbau *et al.* 2009). For the seedling planting study, we calculated the final proportion of surviving seedlings of the pre-grown seedlings that were planted at the onset of the experiment (number of survived seedlings/number of planted seedlings: referred to as ‘*seedling survival*’ hereafter).

On 21 August, we collected the above-ground biomass of the emerged seedlings, and the above- and below-ground biomass of the transplanted seedlings. All biomass was oven-dried at 70 °C for 48 h before weighing. Because the emerged seedlings were still very tiny after 2 months of growth in subarctic conditions, and therefore the handling error large, we only used biomass data of the transplanted seedlings in our analyses.

### Statistical analyses

The design of this study was a split-split plot experiment with the main plots arranged in a randomized complete block design with three random blocks. Fertilization was applied as a split-plot factor and species represented the split-split plot level (Fig. 1). In the analyses, we used mean values of the two observations per species in each subplot to prevent pseudo-replication.

The responses of the recruitment characteristics to the warming and fertilization treatments were tested by means of linear mixed models. Prior to analyses, all recruitment characteristics were square root-transformed and all biomass data log-transformed to obtain a normal distribution and homogeneity of variance. For recruitment, we consecutively tested models with seedling emergence, seedling mortality, seedling establishment and MGT as dependent variables. The models included warming, fertilization, species, and all their two-way and three-way interactions as fixed effects and block, block × warming, block × warming × fertilizer as random effects. For the seedling planting study, similar models were created for shoot, root and total biomass and for survival of the planted seedlings. Here, we additionally included seedling leaf length at the onset of the experiment as a fixed factor to correct for initial differences in seedling size. Differences among means were further analysed by pairwise comparisons, using least significant differences. For ease of interpretation, untransformed values for means are presented in the figures.

To examine if plant-available amounts of nitrogen varied between fertilization and warming treatments we ran two linear mixed models, one for ammonium and one for nitrate. Warming, fertilization and their interaction were included as fixed effects in the model and block and block × warming as random effects. Nutrient concentrations were log-transformed prior to analyses.

We also examined if soil moisture was influenced by the warming treatments. Data were analysed by means of a general linear model with soil moisture as dependent variable and heating, month and block as fixed factors. A Tukey HSD test was used to compare differences between months. All analyses were done with SPSS 21.

## Results

### Environmental measurements

Average ambient surface temperatures (*n* = 3920), representing the seedling environment, were 13.7 ± SD 7.5, 14.1 ± 6.6 and 12.4 ± 6.9 in the three ambient plots, with maximum absolute values of 38.8, 35.6 and 37.1 °C and minimum absolute values of −3.0, −2.2 and −3.3 °C, respectively. By means of the FATI technique, these surface temperatures were continuously elevated by 2.8 ± SD 0.3, 2.9 ± 0.8 and 2.8 ± 0.3 °C, respectively (*n* = 3920, non-contact semiconductor measurements), in the three heated plots of each plot pair. The temperature increase was thus close to the 3 °C we had aimed for.

Air temperatures were hardly affected by the heating treatment (+0.22 ± SD 0.55 °C; averaged over the three plots), whereas soil temperatures were on average increased by 1.6 ± SD 0.4, 1.0 ± 0.3 and −0.1 ± 0.3 °C at 2.5, 7.5 and 15 cm depth, respectively, in the heated compared to the ambient plots. Interestingly, volumetric water content in the top soil layer was slightly higher in the heated (0.45 m^3^ m^−3^) than the ambient (0.40 m^3^ m^−3^) plots (*F*_1, 11798_ = 1292.76, *P* < 0.001; **see Supporting Information—Fig. S1**). It decreased significantly (*F*_2, 11798_ = 2940.99, *P* < 0.001) from June (0.47 m^3^ m^−3^) to July (0.44 m^3^ m^−3^) and August (0.36 m^3^ m^−3^; **see Supporting Information—Fig. S1**). In addition, there was a significant effect of ‘block’ (*F*_2, 11798_ = 2038.29, *P* < 0.001), indicating that soil moisture content was affected by the location of the plot pair.

**Figure 2.**
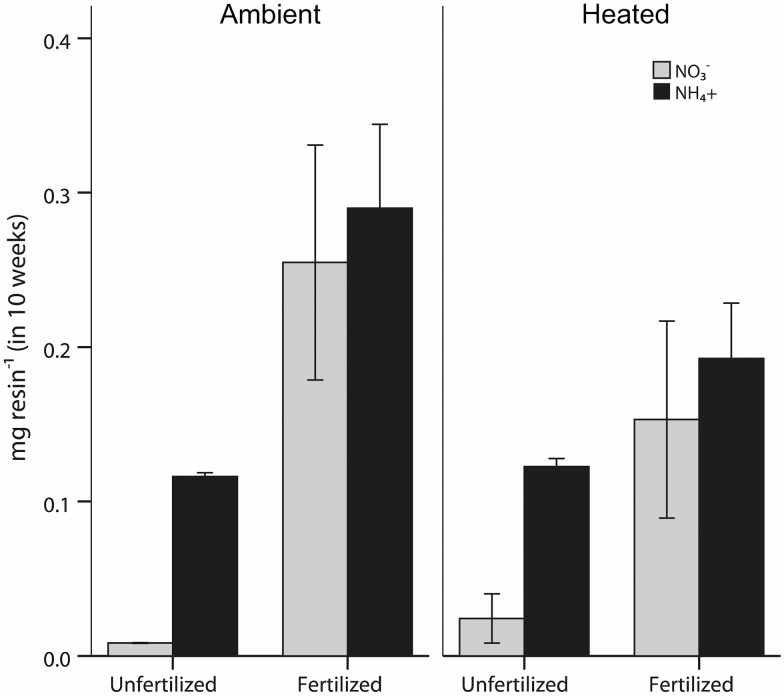
Amounts of nitrate (NO_3_^−^) and ammonium (NH_4_^+^) (means ± 1 SE) absorbed per resin capsule (three resins per plot) over the course of the experiment in the different treatments (ambient, heated, unfertilized and fertilized).

Fertilization resulted in an almost 10-fold increase in plant-available amounts of NO_3_^−^ in the soil, and doubled the availability of NH_4_^+^ (*F*_1, 32_ = 22.28, *P* < 0.001 and *F*_1, 32_ = 43.11, *P* < 0.001 for NH_4_^+^ and NO_3_^−^, respectively), whereas warming had no significant effect on N availability (*F*_1, 32_ = 2.13, *P* = 0.154 and *F*_1, 32_ = 1.20, *P* = 0.282 for NH_4_^+^ and NO_3_^−^, respectively; Fig. 2). The increase in bioavailable nutrients was however more pronounced in the ambient than the heated plots, which was reflected in a nearly significant interaction between warming and fertilization (*F*_1, 32_ = 3.42, *P* = 0.074 and *F*_1, 32_ = 3.62, *P* = 0.066 for NH_4_^+^ and NO_3_^−^, respectively; Fig. 2).

### Effects of warming on germination and seedling establishment

**Figure 3.**
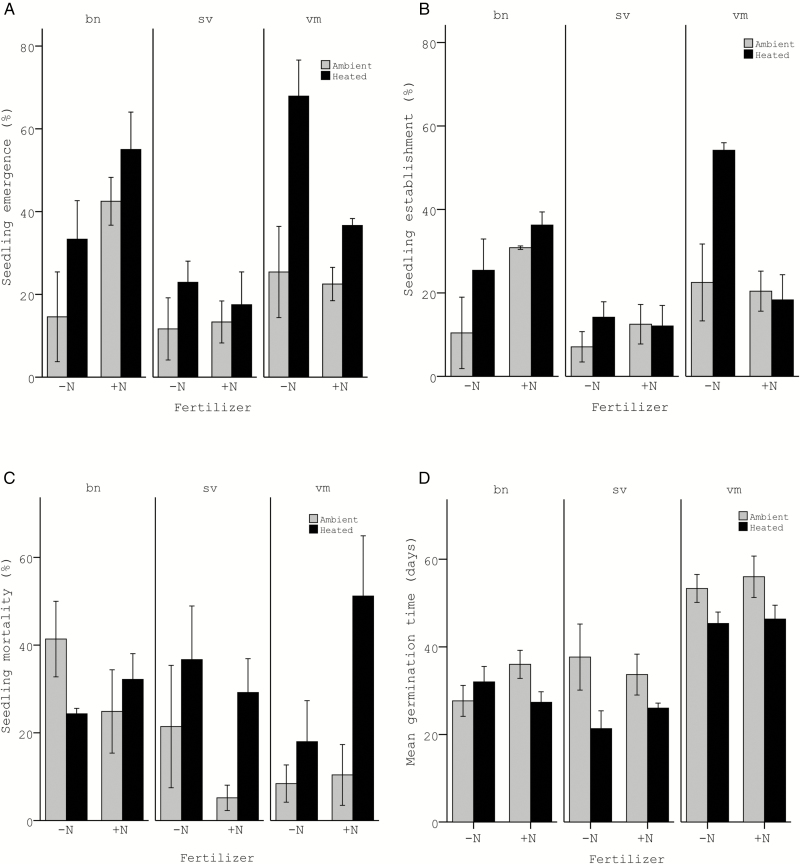
Responses (means ± 1 SE) of (A) seedling emergence, (B) seedling establishment, (C) seedling mortality and (D) MGT of the added seeds (seed sowing study) to the heating and nitrogen addition treatments. Grey bars indicate plots at ambient temperatures, and black bars indicate heated plots (+ 3 °C). ‘−N’ indicates no fertilizer added, and ‘+N’ indicates N addition. bn = *Betula nana*; sv = *Solidago virgaurea*; vm = *Vaccinium myrtillus*.

**Figure 4.**
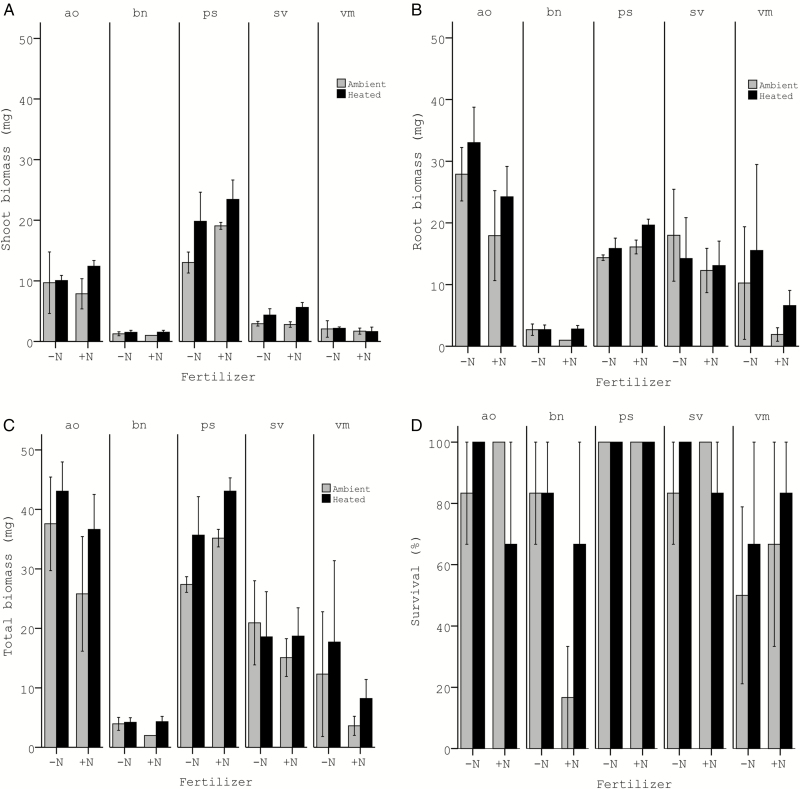
Responses (means ± 1 SE) of (A) shoot biomass, (B) root biomass, (C) total biomass and (D) survival of the transplanted seedlings (seedling planting study) to the heating and nitrogen addition treatments. Grey bars indicate plots at ambient temperatures, and black bars indicate heated plots (+ 3 °C). ‘−N’ indicates no fertilizer added, and ‘+N’ indicates N addition. ao = *Anthoxanthum odoratum*; bn = *Betula nana*; ps = *Pinus sylvestris*; sv = *Solidago virgaurea*; vm = *Vaccinium myrtillus*.

Warming significantly increased seedling emergence (39 % in heated vs. 22 % in ambient plots), independent of species and fertilization treatment, but did not affect seedling establishment in any of the species (Fig. 3A and B; Table 1). The latter was the result of significantly higher seedling mortality rates in the heated compared to the ambient plots (32 % vs. 19 %, respectively; Fig. 3C; Table 1). In the seedling planting study, warming increased above-ground biomass (8.3 mg in heated vs. 6.9 mg in ambient plots; Fig. 4A; Table 2), but had no effect on root biomass (15 mg in heated vs. 13.6 mg in ambient; Fig. 4B; Table 2) nor on total biomass (23.2 mg in heated vs. 20.4 mg in ambient; Fig. 4C; Table 2). Survival of the planted seedlings was not affected by the warming treatment (85 % vs. 78 % in heated and ambient plots, respectively; Fig. 4D; Table 2).

**Table 1.** Effects of warming, fertilization and species identity on seedling emergence, seedling mortality, seedling establishment and MGT of seeds sown in extant subarctic meadow communities at the onset of the treatments. Analyses were performed by means of linear mixed models. *P*-values < 0.05 are in bold. Seedling emergence, seedling mortality and seedling establishment were square root-transformed before analyses.

Source of variation	Seedling emergence		Seedling mortality		Seedling establishment		MGT	
	*F*_d.f._	*P*	*F*_d.f._	*P*	*F*_d.f._	*P*	*F*_d.f._	*P*
Warming (W)	8.83_1, 6_	**0.025**	7.51_1, 6_	**0.034**	4.37_1, 2_	0.172	4.55_1, 2_	0.167
Fertilization (F)	0.48_1, 6_	0.516	0.01_1, 6_	0.918	0.19_1, 4_	0.688	0.49_1, 4_	0.523
Species (S)	16.14_2, 16_	**<0.001**	2.30_2, 16_	0.133	11.80_2, 16_	**<0.001**	66.44_2, 16_	**<0.001**
W × F	2.07_1, 6_	0.200	3.15_1, 6_	0.126	4.94_1, 4_	0.090	0.28_1, 4_	0.627
W × S	1.26_2, 16_	0.311	2.63_2, 16_	0.103	0.54_2, 16_	0.592	3.12_2, 16_	0.072
F × S	9.88_2, 16_	**0.002**	2.12_2, 16_	0.152	9.13_2, 16_	**0.002**	0.09_2, 16_	0.912
W × F × S	0.11_2, 16_	0.900	0.24_2, 16_	0.790	0.55_2, 16_	0.587	3.63_2, 16_	**0.049**

**Table 2.** Effects of warming, fertilization and species identity on shoot, root and total biomass, and on survival of pre-grown seedlings planted in extant subarctic meadow communities at the onset of the experiment. Analyses were performed by means of linear mixed models. *P*-values < 0.05 are in bold. All biomass data were ln-transformed before analyses. To correct for differences in initial seedling size at the time of planting, leaf length was added as a fixed factor in the model.

Source of variation	Shoot biomass		Root biomass		Total biomass		Seedling survival	
	*F*_d.f._	*P*	*F*_d.f._	*P*	*F*_d.f._	*P*	*F*_d.f._	*P*
Warming (W)	8.19_1, 7_	**0.023**	1.20_1, 4_	0.335	1.89_1, 4_	0.242	0.43_1, 4_	0.548
Fertilization (F)	0.19_1, 7_	0.676	3.16_1, 26_	0.087	2.38_1, 26_	0.135	0.07_1, 27_	0.792
Species (S)	44.24_4, 25_	**<0.001**	10.87_4, 27_	**<0.001**	16.15_4, 27_	**<0.001**	2.88_4, 28_	**0.041**
W × F	0.16_1, 8_	0.697	1.88_1, 26_	0.182	0.78_1, 26_	0.384	0.50_1, 27_	0.487
W × S	0.83_4, 24_	0.517	1.07_4, 26_	0.393	0.87_4, 27_	0.495	0.53_4, 27_	0.715
F × S	0.42_4, 24_	0.795	0.59_4, 26_	0.672	0.72_4, 26_	0.589	0.12_4, 27_	0.973
W × F × S	0.82_4, 24_	0.528	0.84_4, 26_	0.512	0.62_4, 26_	0.656	1.90_4, 27_	0.138
Leaf length	19.88_1, 29_	**<0.001**	5.18_1, 27_	**0.031**	8.38_1, 28_	**0.007**	5.40_1, 30_	**0.027**

The species differed in germination time, with *V. myrtillus* germinating significantly later than the other two species (Fig. 3D; MGT = 30, 31 and 50 days for *S. virgaurea*, *B. nana* and *V. myrtillus*, respectively). Analyses per species (significant warming × fertilization × species interaction; Table 1) indicated that warming reduced the germination time in *S. virgaurea*, from 36 to 24 days (*F*_1, 4_ = 7.86, *P* = 0.049), and that there was a similar pattern in *V. myrtillus* (from 55 to 46 days), albeit not significant (*F*_1, 4_ = 4.261, *P* = 0.109). For *B. nana*, no effect of warming on MGT could be detected (*F*_1, 4_ = 0.256, *P* = 0.640).

### Effects of nitrogen addition on germination and seedling establishment

The effects of fertilization on recruitment were species-specific and independent of warming (Fig. 3A and B; Table 1). Fertilization improved both seedling emergence and establishment in *B. nana* (*F*_1, 15_ = 10.85, *P* = 0.005 and *F*_1, 13_ = 8.50, *P* = 0.012 for emergence and establishment, respectively), had no effect in *S. virgaurea* (*F*_1, 15_ = 0.044, *P* = 0.835 and *F*_1, 13_ = 0.144, *P* = 0.710) and reduced seedling establishment in *V. myrtillus* (*F*_1, 15_ = 2.256, *P* = 0.154 and *F*_1, 13_ = 5.722, *P* = 0.032). N addition did neither influence seedling mortality, nor the timing of germination (Fig. 3C and D; Table 1).

N addition, in contrast to warming, had no effect on any of the biomass measures in the seedling experiment (Fig. 4; Table 2), neither was survival of the planted seedlings influenced by fertilization. Survival depended only on species and initial seedling size (Table 2). Whereas all seedlings of *P. sylvestris* survived, *B. nana* and *V. myrtillus* had the lowest survival rates with 62.5 % and 66.7 % survival, respectively.

## Discussion

### Effects of warming on germination and seedling establishment

Seedling emergence increased in response to a temperature rise of 2.8 °C, which was consistent with our expectations and with several lab studies (Bell and Bliss 1980; Graae *et al.* 2008; Milbau *et al.* 2009; Müller *et al.* 2011). Surprisingly, this positive effect was counteracted by higher seedling mortality in the heated compared to the ambient plots, resulting in no net warming effect on seedling establishment. As warming generally stimulates growth in arctic and alpine species (e.g. Parsons *et al.* 1994; Arft *et al.* 1999) we would have expected the opposite. Potentially, the observed higher mortality in the heated plots was the result of an indirect, rather than a direct effect of warming. The most obvious option would be drought, but the measured higher moisture content in the topsoil layer (upper 5 cm) of the heated compared to the ambient plots **[see Supporting Information—Fig. S1]** refutes this, although the possibility of drought directly at the soil surface cannot be excluded. Surface drought could be especially important straight after seedling emergence. Other alternatives are improved conditions for pathogens (Bebber *et al.* 2013) or increased competition, e.g. for light and nutrients, from the resident vegetation (Klanderud and Totland 2007; Olsen and Klanderud 2014). The latter is very likely as moderate warming generally increases above-ground production and plant height in tundra communities (Walker *et al.* 2006).

Although warming neither influenced first-year seedling establishment, nor the survival of planted seedlings, it did improve seedling growth (Fig. 4). This was according to our expectations, and could be important for over-winter survival. Due to the short growing season in arctic regions (~3 months in Abisko; Molau *et al.* 2005), gaining critical biomass and sufficient carbon reserves during the first growing season is essential to survive the long-lasting and harsh winter, and thus reach the following growing season (Stocklin and Baumler 1996; Schütz 2002). Seedlings grown under warmer conditions, thereby reaching higher biomass, are accordingly expected to have higher survival rates. Similarly, we expect that the observed earlier germination in *S. virgaurea* and *V. myrtillus* as a response to warming, and the therefrom resulting longer growing season, will improve these species’ chances for long-term survival. However, due to the short duration of our experiment, we should be careful with drawing long-term conclusions.

### Effects of nitrogen addition on germination and seedling establishment

Responses to fertilization were species-specific. Nitrogen addition had a positive effect on both seedling emergence and seedling establishment in *B. nana*, no effect in *S. virgaurea* and a negative effect in *V. myrtillus*. The potential role of nitrogen, and especially nitrate, as a stimulator of seed germination is known (Bewley and Black 1982; Hilhorst and Karssen 1992; Baskin and Baskin 1998), but to our knowledge the mechanisms of action still need to be elucidated and studies thus far have mainly focussed on commercially interesting plant species. Consequently, we cannot explain why the three species used in this study showed contrasting germination responses to nitrogen fertilization and argue that more research on the role of nutrients for seedling emergence in arctic and alpine ecosystems is needed.

The finding that fertilization with realistic nitrogen levels affected seedling establishment of *B. nana* and *V. myrtillus*, two co-dominant deciduous dwarf shrubs in subarctic tundra, in opposite ways, may have important consequences for future community development, as better seedling establishment under richer soil conditions could help *B. nana* dominate over *V. myrtillus* under future conditions. This result is in accordance with data from observational studies showing a substantial increase in abundance of *B. nana* in subarctic tundra over the last decades (Olofsson *et al.* 2009; Rundqvist *et al.* 2011) in contrast to no changes in *V. myrtillus* (Olofsson *et al.* 2009).

Contrary to our expectations, fertilization did not increase seedling biomass. This may be related to the relatively low amounts of nitrogen added. Whereas fertilization studies in arctic environments regularly use amounts of up to 10 g N m^−2^ (e.g. Michelsen *et al.* 1996; Press *et al.* 1998), which typically result in clear plant responses, we only added 1.4 g m^−2^ to simulate more realistic changes in N availability (see also Zamin and Grogan 2012). Potentially, the young seedlings failed to successfully take up the added N in competition with the soil microbial community and the extant vegetation in this N-limited tundra ecosystem (Mack *et al.* 2004; Elser *et al.* 2007). Alternatively, increased N uptake by the seedlings could have resulted in higher tissue N concentrations (not measured) rather than growth, as is regularly seen in high-latitude regions, where biomass responses of plants to N addition are often limited (Xia and Wan 2008).

We did not observe an interactive effect among warming and N addition in this study. We had expected a stronger fertilization effect in the heated compared to the ambient plots because increased plant biomass in response to warming should increase plant N demand (An *et al.* 2005). However, the complete lack of any N effect on seedling growth suggests either that seedling growth was not N-limited, or that the seedlings could not access the added N, but the exact reason cannot be revealed here. Interestingly, plant-available nutrients (measured by means of resin capsules) in the fertilization treatment were lower in the heated than ambient plots (Fig. 1) suggesting an increased uptake of the added N by the extant plants and/or the soil microbial community in the heated plots.

### Implications of our findings

In general, neither warming, nor N addition, had a strong positive effect on seedling establishment in this tundra ecosystem. Although seedlings obtained higher above-ground biomass when grown in a warmer environment, none of the treatments increased the number of seedlings by the end of the summer. The only exception was *B. nana*, in which fertilization, but not warming, resulted in more seedlings.

The lack of a general increase in seedling establishment under warmer and/or more fertile conditions may have important consequences for future tundra community composition. The current low recruitment numbers in these ecosystems (Bell and Bliss 1980; Graae *et al.* 2011; Müller *et al.* 2011; Milbau *et al.* 2013), and the lack of improvement under future environmental conditions as indicated in this study, suggest that many species common to subarctic environments will only be able to migrate slowly, due to poor germination and seedling establishment. If colonization by recently expanding or invading species occurs faster than resident species are able to disperse to new sites, competitively weak species might disappear due to the increased role of competition from the newcomers (Grabherr *et al.* 1995; Gottfried *et al.* 1999; Kueffer *et al.* 2013). This already seems to happen in certain alpine areas, where a number of alpine species have decreased in abundance, although plant species richness has generally increased (Klanderud and Birks 2003). Similarly, in subarctic ecosystems, alien species have been shown to successfully invade alpine plant communities (Lembrechts *et al.* 2014). To better understand why temperate species are successful in these cold environments, an interesting focus for future studies could be whether recruitment success in cold areas and its response to warming differs between arctic, boreal and temperate species, the latter generally being better adapted to sexual reproduction.

Our findings may also be informative for species distribution models. The current study shows empirically that in (sub)arctic environments, even under warmer and more nutrient-rich conditions, establishment from seed remains limited. The prediction of future species distributions solely based on abiotic factors (Guisan and Zimmermann 2000; Randin *et al.* 2009) may therefore overestimate future species’ ranges, not taking into account poor plant establishment in cold regions. The inclusion of realistic establishment values in models for predicting range shifts in arctic environments may be a way to overcome this. Moreover, we suggest using ‘establishment limitation’ instead of ‘dispersal limitation’ (Engler *et al.* 2009) in relation to arctic plant migration, given that the mean dispersal distance estimated for arctic plants is 570 km (Hoffmann 2012), whereas establishment seems to be a larger bottleneck.

Lastly, we found important interspecific differences in germination and establishment responses to fertilization. As these concern dominant species in subarctic regions, i.e. *B. nana* and *V. myrtillus*, and we currently cannot explain why they responded differently, we argue for more research into the role of fertilization for germination and seedling establishment of northern species. The observed interspecific differences should also be taken into account when making predictions of (sub)arctic community responses to a changing climate.

## Sources of Funding

This work was funded by a grant from the Special Research Fund (BOF/KP 21954) of the University of Antwerp to A. Milbau, and by the Research Foundation Flanders (FWO) by means of a postdoctoral fellowship to A. Milbau.

## Contributions by the Authors

A.M. and I.N. designed the study; A.M. and N.V. collected and analysed the data; F.K. and I.N. developed the FATI technique and set up the equipment in the field; A.M. wrote the manuscript with support from I.N.

## Conflicts of Interest

None declared.

## Supporting Information

The following additional information is available in the online version of this article—

Figure S1. Volumetric soil water content (means ± 1 SE) in the ambient and the heated treatments presented per month.

## Supplementary Material

Fig-S1
