# Supplementary figures and images for: Both seed germination and seedling mortality increase with experimental warming and fertilization in a subarctic tundra
Source: AoB Plants. 2017 Sep 1;9(5):plx040. doi: 10.1093/aobpla/plx040 (PMC5629451; doi:10.1093/aobpla/plx040)

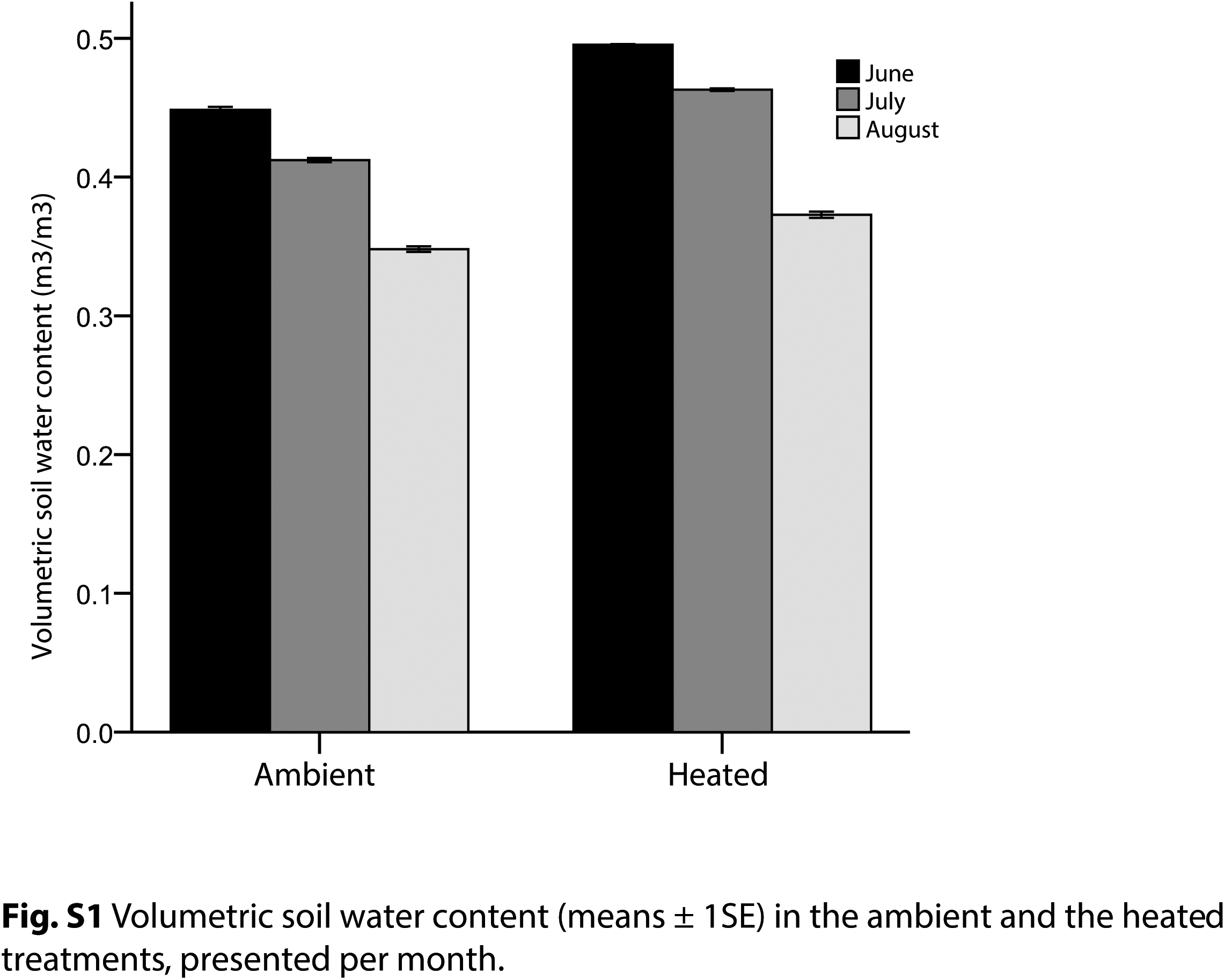

Supplement: Fig-S1 [file plx040_suppl_Fig-S1.png]
